# Clinical relevance of peroxisome proliferator-activated receptor-gamma expression in myxoid liposarcoma

**DOI:** 10.1186/s12885-016-2524-6

**Published:** 2016-07-11

**Authors:** Akihiko Takeuchi, Norio Yamamoto, Toshiharu Shirai, Katsuhiro Hayashi, Shinji Miwa, Seiichi Munesue, Yasuhiko Yamamoto, Hiroyuki Tsuchiya

**Affiliations:** Department of Orthopaedic Surgery, Kanazawa University Graduate School of Medical Sciences, 13-1 Takara-machi, Kanazawa, 920-8641 Japan; Department of Orthopaedic Surgery, Kyoto Prefectural University of Medicine, 465 Kajii-cho, Kawaramachi-Hirokoji, Kamigyo-ku, Kyoto, 602-8566 Japan; Department of Biochemistry and Molecular Vascular Biology, Kanazawa University Graduate School of Medical Sciences, 13-1 Takara-machi, Kanazawa, 920-8640 Japan

**Keywords:** Peroxisome proliferator-activated receptor gamma, Myxoid liposarcoma, Immunohistochemistry, PCR, Prognostic marker

## Abstract

**Background:**

Peroxisome proliferator-activated receptor gamma (PPARγ) is a ligand-activated transcription factor that belongs to the nuclear hormone receptor superfamily. PPARγ is essential in adipocyte differentiation from precursor cells. Its antitumorigenic effects are reported in certain malignancies; however, its effects in liposarcoma are unclear.

**Methods:**

We analyzed PPARγ expression using immunohistochemistry (IHC) in 46 patients with myxoid liposarcoma [MLS; median age, 47 years (range, 14–90 years) and mean follow-up period, 91 months (range, 13–358 months)]. *PPARγ* mRNA expression levels were measured by quantitative reverse transcription polymerase chain reaction. Further, we evaluated the correlation of PPARγ expression with clinical outcomes.

**Results:**

We found that the metastasis-free survival rate was significantly higher in lower PPARγ expressers [34 patients with labeling index (LI) <50 %] than in higher expressers (12 patients with LI ≥50 %; *p* = 0.01). Cox multivariate analysis revealed that a higher PPARγ level was an independent predictor of metastasis (relative risk = 6.945, *p* = 0.026). Furthermore, using 28 fresh MLS specimens, we confirmed an increased *PPARγ* mRNA expression level in the higher LI group (*p* = 0.001).

**Conclusions:**

In this study, higher PPARγ expression in MLS was a risk factor associated with distant metastasis; therefore, it would be a novel prognostic marker for MLS. Further analyses will help to understand the correlation between PPARγ expression and tumor malignancy in liposarcoma.

## Background

Liposarcoma is one of the most common adult soft tissue sarcomas, accounting for 15–20 % of all sarcomas [1]. It is histologically classified into 3 subtypes: dedifferentiated, myxoid, and pleomorphic liposarcomas. Of these, myxoid liposarcoma (MLS) accounts for one-third to one-half of liposarcomas [2]. A diagnostic nomenclature of “round cell liposarcoma” was used when the round cell component of MLS tissues was >5 % [3]. However, MLS and round cell liposarcoma were found to represent the same entity because they share a key chromosomal translocation t(12;16)(q13;p11), generating a fusion oncogene *FUS-DDIT3* [4, 5]. Recent evidence has indicated that the activation of PI3K/Akt pathway via activating mutation of PIK3CA, loss of PTEN, or IGF1R expression would have a role in round cell transformation [6]. Although MLS is considered to be a low-to-intermediate grade malignancy [1], distant metastasis of the tumor cells may occasionally occur. It is currently believed that a proportion of round cells is an established predictor of clinical outcome in patients with MLS. For example, MLS containing >10 % of round cells may indicate poor prognosis because of the high risk of metastasis occurrence [1]. However, there is yet no consensus regarding the percentage of round cells that would help in the grading of MLS. Furthermore, the benefit of chemotherapy is yet controversial in the treatment of MLS [7, 8]. Therefore, additional and/or stronger prognostic markers are required to accurately predict prognosis and to develop effective therapeutic strategies for patients with MLS.

Peroxisome proliferator-activated receptor gamma (PPARγ) is a master regulator of adipocyte differentiation [9] and is expressed in various types of cancers, such as breast [10], colon [11], prostate [12], thyroid cancers [13], and giant cell tumor of bone [14]. A significant elevation in PPARγ expression was reported in MLS, pleomorphic liposarcoma, and dedifferentiated liposarcoma, particularly in differentiated areas of dedifferentiated liposarcoma, compared with lipoma or well-differentiated liposarcoma [15]. However, a correlation between PPARγ expression and clinical outcomes of MLS has not been yet completely elucidated. Therefore, this study aimed to evaluate PPARγ expression in MLS and elucidate whether PPARγ expression could be a prognostic biomarker in the recurrence and metastases of MLS.

## Methods

### Patients and tumor specimens

Patients with MLS were enrolled by searching the hospital computer database, to find who had been treated at the Department of Orthopaedic Surgery in Kanazawa University Hospital between 1989 and 2012. Forty-six patients with MLS comprised the cohort of the current study. The median age was 47 years (range, 14–90 years), and the mean follow-up period was 91 months (range, 13–358 months). Thirty-eight patients had primary lesions, and 8 patients presented with recurrent tumors. According to the American Joint Committee on Cancer classification [16], 9, 2, and 35 patients were classified as stage IIA, IIB, and III, respectively. The primary tumor sites were in the upper extremity (2 cases), lower extremity (38 cases), and axial location (6 cases). Thirty-eight patients had no round cell component. Seven tumors contained <5 % of the round cell component and only 1 tumor showed >5 % of the round cell component. Paraffin-embedded tissue specimens of surgical resected primary or recurrent tumors from the current 46 patients and 28 of 46 frozen tumor specimens were available for immunohistochemistry (IHC) and quantitative reverse transcription (RT)-polymerase chain reaction (PCR) analyses, respectively. The study was approved by the Ethics Committee for Medical Studies at the Kanazawa University Graduate School of Medical Sciences.

### Immunohistochemical analysis and scoring

Tissue specimens were fixed in 20 % formalin and embedded in paraffin. They were retrieved from the surgical pathology files of the Pathology Section of Kanazawa University Hospital (Kanazawa, Japan). For each case, one representative block of formalin-fixed and paraffin-embedded tumor tissue was selected. All sections were cut at 4-μm thickness for IHC. A mouse monoclonal antibody against PPARγ (1:250, sc-7273, Santa Cruz Biotechnology, Santa Cruz, CA, USA) was used as the primary antibody and anti-mouse IgG conjugated with peroxidase-labeled polymers (EnVision, Dako, Carpinteria, CA, USA) was used as a secondary antibody. After visualization of the reaction product, sections were counterstained with Meyer’s hematoxylin and coverslipped for microscopic observation. Apparent brown stains were considered to be immunopositive spots. Negative controls were used by excluding the primary antibody. All positive and negative cells were counted in a minimum of 5 non-overlapping visual fields at 200× magnification. The labeling index (LI) for PPARγ was calculated as the percentage of positive cells among the total number of cells counted, which was at least 250 tumor cells [17]. LI was performed by two assessors (AT and SM) blinded to patient outcome and the assessment was duplicated. With this evaluation, we categorized higher and lower PPARγ expressers as those with > and <50 % of LI, respectively.

### Real-time reverse transcription polymerase chain reaction

Twenty-eight frozen tumor tissues were available for real-time RT-PCR analysis. Total mRNA was isolated using the RNeasy Mini Kit (Qiagen, Hilden, Germany) according to the manufacturer’s instructions. The quantity of RNA was measured using the NanoDrop lite (Thermo Fisher Scientific Inc., Waltham, MA, USA). First-strand cDNA was generated from total RNA using the QuantiTect Reverse Transcription Kit (Qiagen) with a poly (dT) oligonucleotide primer. *PPARγ* (QT00029841) and *GAPDH* (QT01192646) primers were purchased from Qiagen. Real-time PCR was performed using QuantiFast SYBR Green PCR Kit (Qiagen) and Stratagene Mx3000P QPCR System (Agilent Technologies, La Jolla, CA, USA). The relative mRNA expression level was calculated using a comparative Ct (ΔCt) method with LinReg PCR software (http://LinRegPCR.nl).

### Statistical analysis

Statistical analysis was performed using SPSS v.19.0 (SPSS Inc., Chicago, IL, USA). Correlation of the PPARγ LI with patient prognosis and histological subtype (pure type vs. round cell group) was evaluated using the chi square test. The following demographic and treatment factors were examined for prognostic importance: patient age, sex, tumor site (extremity or axial), tumor size, patient status (primary or recurrence), presence of round cell component, surgical margin, chemotherapy, radiotherapy, and PPARγ LI. The association of each factor with subsequent tumor recurrence and distant metastasis was analyzed using the log-rank test and Cox proportional hazards regression analysis using backward step-by-step exclusion. In Cox proportional hazards models, the factors with *p* < 0.3 in univariate analysis were included. The relative *PPARγ* mRNA and protein expressions were statistically evaluated using Student’s *t*-test. For each test, results were considered statistically significant whenever a probability (*p*) value <0.05 was achieved.

## Results

**Fig. 1:**
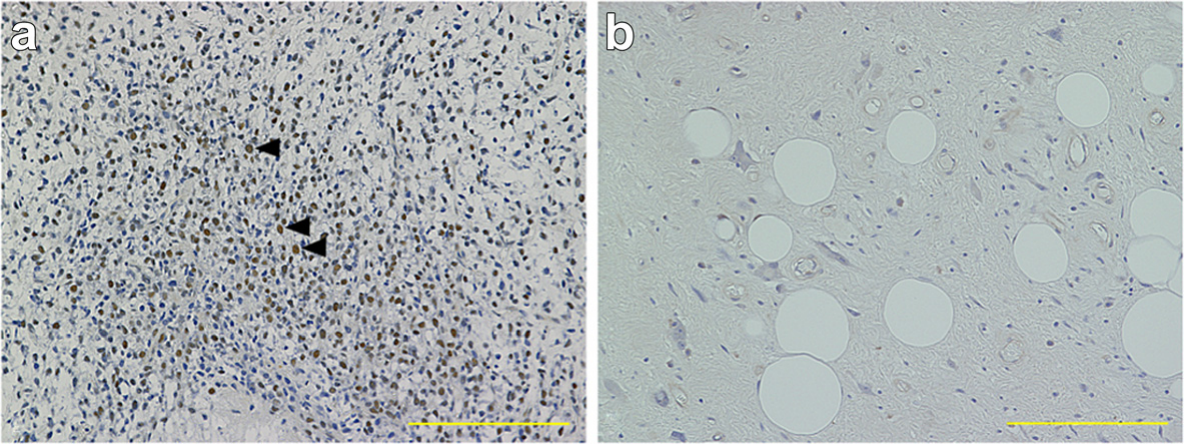
**a** Higher PPARγ expression was observed using immunohistochemistry [IHC; labeling index (LI) ≥50 %]. Positive signals were detected in the nucleus. This section contains a round cell component and most round cells were positive for PPARγ (black arrow head). **b** Lower PPARγ expression (LI <50 %). The scale bar corresponds to 200 μm

**Fig. 2:**
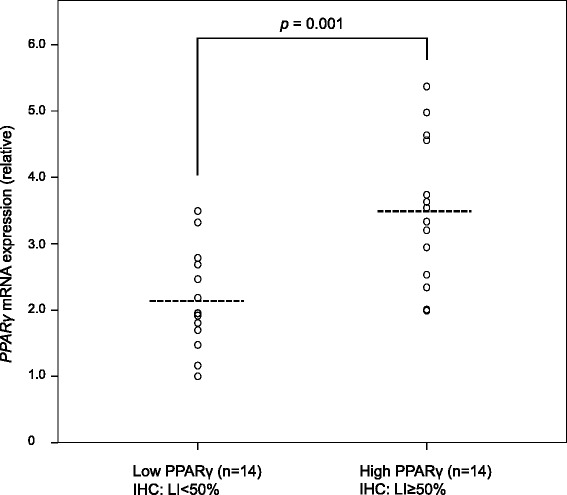
*PPARγ* mRNA expression levels. The relative *PPARγ* mRNA expression in the specimens from patients with higher PPARγ expression (LI ≥50 %) was 3.48 ± 0.29 (the mean and standard error). The value of lower PPARγ expression (LI <50 %) was 2.13 ± 0.20. There was a significant difference in *PPARγ* mRNA levels between the two groups (*p* = 0.001). Dashed lines represent the mean value

**Fig. 3:**
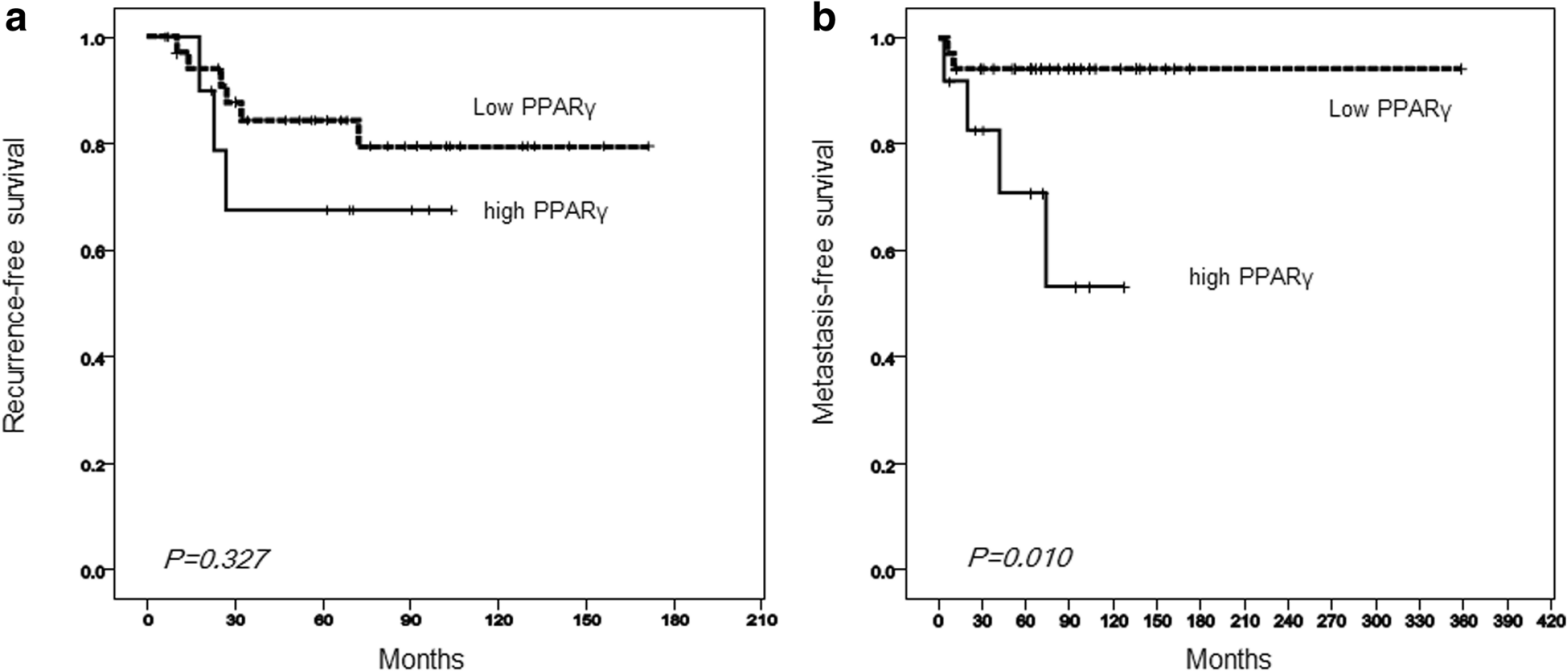
**a** Recurrence-free survival rate. There were no significant differences in recurrence-free survival between patients with higher and lower PPARγ expression (*p* = 0.327). **b** Metastasis-free survival rate. Metastasis-free survival rate was significantly higher in patients with lower PPARγ expression than in those with higher PPARγ expression (*p* = 0.01)

**Fig. 4:**
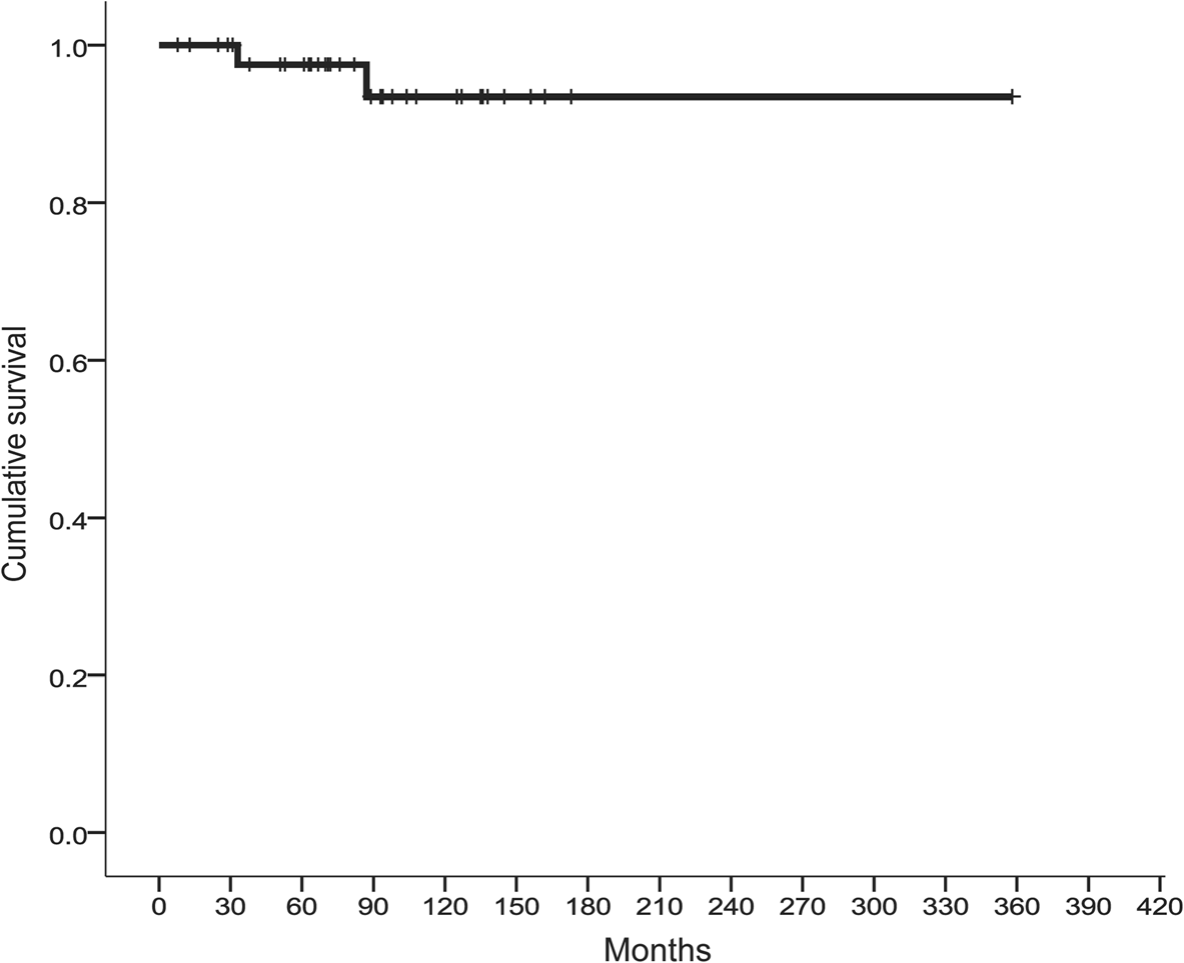
The overall cumulative 5- and 10-year survival rates. The 5- and 10-year survival rates were 97.5 and 93.4 %, respectively

We first examined the correlation between the clinical outcomes and PPARγ expression by IHC in tumors from patients with MLS. Positive signals for PPARγ were obtained in the nucleus of the tumor cells (Fig. 1a). The higher (LI ≥50 %) and lower (LI <50 %) expressions of PPARγ were observed in the specimens from 12 and 34 patients with MLS (Fig. 1a, b), respectively. Among the 34 samples, an absence of PPARγ expression in the tumor was demonstrated in 5 MLS patients. We confirmed *PPARγ* mRNA expression levels by quantitative RT-PCR. The relative *PPARγ* mRNA levels were found to be 3.48 *vs* 2.13 in tumors in patients with higher *vs* lower PPARγ expression by IHC, respectively (*p* = 0.001; Fig. 2). Chi square analysis showed that PPARγ expression was not significantly associated with patient status of MLS recurrence (*p* = 0.178; Table 1). Among 8 patients with MLS with the round cell component, 4 patients were found to be higher expressers of PPARγ and the remaining 4 had lower PPARγ expression (Table 2). Furthermore, in the group with no round cells, 8 patients had higher PPARγ expression and the remaining 30 patients had lower expression. However, there were no statistically significant differences in PPARγ expression between the 2 groups (*p* = 0.178; Table 2). Most patients with round cells showed positivity for PPARγ (Fig. 1a). Local recurrence occurred in 9/46 (19.6 %) patients with MLS at our institute (Table 3). Time to local recurrence varied from 10 to 72 months. In a univariate analysis, extremity of the MLS sites (*p* = 0.001), primary tumor (*p* < 0.001) and negative surgical margin (*p* = 0.001) significantly correlated with a better recurrence-free survival (Table 3). The 5-year local recurrence-free survival rate was 83.8 and 67.5 % in the PPARγ lower and higher expressers, respectively (Table 3). However, PPARγ expression level was not significantly associated with local recurrence (*p* = 0.327; Fig. 3a). Using a multivariate analysis, no independent factors were associated with local recurrence. In a total of 46 patients with MLS, 6 (13.0 %) cases developed distant metastasis (Table3), and the time course to distant metastasis varied from 4 to 74 months. The sites of metastases of MLS included the spine (2 cases), the femur (1 case), the retroperitoneum (1 case), the axilla (1 case), and the lung (1 case). The 5-year distant metastasis-free survival rates were 94.1 and 70.1 % in PPARγ lower and higher expresser groups, respectively (Table 3), and there was a statistically significant difference between the lower and higher groups (*p* = 0.01; Fig. 3b). High PPARγ expression was an independent risk factor of distant metastasis of MLS using a multivariate analysis (HR, 6.945; 95 % CI, 1.265–38.15, *p* = 0.026; Table 4). In this study, we could not identify any positive prognostic factors for overall survival using either univariate or multivariate analyses in patients with MLS whose overall cumulative 5- and 10-year survival rates were 97.4 and 92.3 %, respectively (Table 3, Fig. 4).

**Table 1:** Chi-square analysis of PPARγ with status

		Primary	Recurrence	*p* value
PPARγ	low (LI < 50 %)	30	4	
	high (LI ≥ 50 %)	8	4	*p* = 0.178

**Table 2:** Chi-square analysis of PPARγ with or without round cell component

		No round cell	Round cell	*p* value
PPARγ	low (LI < 50 %)	30	4	
	high (LI ≥ 50 %)	8	4	*p* = 0.178

**Table 3:** Univariate analysis of prognostic factors

Factors	No. of patients (event)	5-y RFS	*p* value	No. of patients (event)	5-y MFS	*p* value	No. of patients (event)	5-y OS	*p* value
Overall	46 (9)			46 (6)			46 (2)		
Age									
<45 y.o.	20 (6)	.739	.164	20 (2)	.900	.605	20 (1)	.947	.877
≥45 y.o.	26 (3)	.869		26 (4)	.873		26 (1)	1.000	
Gender									
Male	28 (8)	.736	.101	28 (4)	.888	.898	28 (2)	.962	.264
Female	18 (1)	.933		18 (2)	.889		18 (0)	1	
Site									
Extremity	42 (6)	.839	.001	42 (5)	.898	.479	42 (1)	1	.045
Axial	4 (3)	.500		4 (1)	.750		4 (1)	.750	
Size									
<10 cm	21 (4)	.800	.797	21 (2)	.950	.437	21 (1)	1.000	.857
≥10 cm	25 (5)	.821		25 (4)	.836		25 (1)	.950	
Status									
Primary	38 (4)	.886	<.001	38 (4)	.892	.378	38 (1)	.970	.268
Recurrent	8 (5)	.429		8 (2)	.857		8 (1)	1.000	
Round cell									
No	38 (6)	.856	.092	38 (4)	.921	.216	38 (2)	.970	.551
Yes	8 (3)	.571		8 (2)	.686		8 (0)	1	
Surgical margin									
Negative	39 (4)	.884	<.001	39 (4)	.922	.248	39 (1)	1	.216
Positive	7 (5)	.429		7 (2)	.714		7 (1)	.857	
Chemotherapy									
No	32 (8)	.758	.173	32 (5)	.867	.485	32 (2)	.964	.359
Yes	14 (1)	.917		14 (1)	.929		14 (0)	1	
Radiotherapy									
No	41 (8)	.811	.846	41 (6)	.870	.361	41 (2)	.971	.629
Yes	5 (1)	.800		5 (0)	1		5 (0)	1	
PPARγ									
low (LI < 50 %)	34 (6)	.845	.327	34 (2)	.941	.010	34 (1)	.968	.383
high (LI ≥ 50 %)	12 (3)	.675		12 (4)	.707		12 (1)	1	

*RFS* recurrence-free survival, *MFS* metastasis-free survival, *OS* disease specific overall survival

**Table 4:** Cox proportional hazards regression analysis of factor affecting metastasis-free survival

Factor	Before step-by-step exclusion					After step-by-step exclusion				
	Wald Statistic	Regression coefficient (B)	Relative risk (e^B^)	95 % CI	*p* value	Wald Statistic	Regression coefficient (B)	Relative risk (e^B^)	95 % CI	*p* value
Round cell	0.005	0.08	1.083	0.129–9.084	0.941					
Surgical margin (positive)	1.588	1.321	3.746	0.480–29.228	0.208					
High PPARγ expression (LI ≥ 50 %)	4.791	2.119	8.327	1.248–55.55	0.029	4.972	1.938	6.945	1.265–38.15	0.026

## Discussion

In the present study, we examined the PPARγ expression in tumor cells of patients with MLS by IHC and confirmed *PPARγ* mRNA expression levels using quantitative RT-PCR (Fig. 2). We found that PPARγ expression in tumors was significantly associated with the development of distant metastasis, but was not correlated with recurrence-free or overall survival, in patients with MLS (Fig. 3, Tables 3 and 4). Therefore, we suggest that high PPARγ expression could be a novel risk factor for distant metastasis of MLS.

Tontonoz et al. first reported that PPARγ- and retinoid X receptor-specific ligands stimulated cellular differentiation in liposarcoma cells [18]. In another study, Tajima et al. reported higher PPARγ expression observed in MLS, pleomorphic liposarcoma, dedifferentiated liposarcoma, and the differentiated area of dedifferentiated liposarcoma compared with lipoma and well-differentiated liposarcoma by IHC [15]. However, the correlation and significance of PPARγ expression and clinical outcomes in patients with MLS have not yet been completely evaluated.

In the present study, the incidence of local recurrence of MLS was 19.6 %, which is in the range from 8 to 33 % demonstrated in other reports [3, 18, 19]. In addition, the incidence of distant metastasis in this study was 13.0 %, and this value was within previously reported ranges from 10 to 38 % [19, 20]. It is reported that potential risk factors for clinical outcomes in patients with MLS are (1) tumor size (>10 cm) [21], (2) age (>45 years) [20, 21], (3) presence of the round cell component [2], and (4) non-extremity lesions [20]. In our study, univariate analysis showed that extremity lesion site of the tumor significantly correlated with a better recurrence-free and overall survival (Table 3). However, we could not find any statistically significant correlations using the multivariate analysis. However, PPARγ expression was significantly associated with the distant metastasis-free survival rate by both the univariate (*p* = 0.01; Table 3) and multivariate (*p* = 0.041; Table 4) analyses.

MLS metastases to extrapulmonary sites have been reported in some cases [22]. In this study, 5 of 6 patients developed extrapulmonary site metastasis including the spine (2 cases), the femur (1 case), the retroperitoneum (1 case) and the axilla (1 case). However, the disease-specific deaths occurred only in 2 patients with spine or femur metastasis, which seemed to be a better survival rate when compared to previous reports. Asano et al. reported that the low histological grade was significantly associated with extrapulmonary metastasis [23]. They also reported that the overall survival rate was significantly better for patients with extrapulmonary metastases (63 %) compared to those with pulmonary metastases (0 %) [23].

PPARγ is reported to possess an antitumor activity through the suppression of tumor proliferation and invasion [24] and the induction of differentiation and apoptosis in cancer cells [25]. Therefore, we initially hypothesized that less PPARγ expression may be associated with an aggressive behavior or a shorter survival rate in patients with MLS, which is closely associated with adipocyte differentiation. However, the results were contradictory to our initial expectation. Lower PPARγ expression was significantly associated with being free from distant metastasis of MLS (Table 3, Fig. 3b). Thus, it is yet unknown which molecular mechanisms influence PPARγ expression on MLS tumor malignancy. With regard to our data, higher PPARγ expression is correlated with shorter survival in pancreatic ductal adenocarcinoma [26] and prostate cancer [27] and with the onset and progression of ovarian cancer [28]. Synthetic PPARγ stimulators such as thiazolidinediones (TZDs) are widely used for the treatment of patients with type 2 diabetes mellitus [29]. TZDs have been used to treat liposarcoma patients; however, some clinical trials failed to show favorable results [18, 30, 31]. Experimentally, Pérez-Losada et al. showed the induction of liposarcomas in *FUS-DDIT3* transgenic mice [32]. Although PPARγ expression was significantly expressed in the tumor, adipocytic development was inhibited [32]. Thus, the authors speculated that the downstream signaling of PPARγ in tumor cells would be specifically dysregulated by the *FUS-DDIT3* transgene even at higher PPARγ levels [32]. Therefore, the upregulation of PPARγ expression may be caused by the blockade of downstream signaling of PPARγ in MLS cells. The higher expressers would then be categorized as a malignant phenotype with distant metastasis in MLS. Further detailed studies are required to reveal this possibility. Recently, a novel drug, trabectedin, was developed and introduced as chemotherapy for patients with MLS showing favorable effects [33]. The drug is reported to induce the maturation of MLS lipoblasts in vivo by targeting the *FUS-DDIT3* chimera [34], thus possibly preventing the inhibition of PPARγ signaling. In a phase II clinical trial study of trabectedin for advanced MLS, Gronchi et al. showed that 7 of 29 patients achieved partial response (objective response rate, 24 %; 95 % CI, 10–44 %) [33]. These accumulating data suggest that the PPARγ signaling pathway could be important for carcinogenesis, cell differentiation, and the biology underlying MLS.

Demicco et al. reported the data about the activation of PI3K/Akt pathway and mutation analysis of *PIK3CA* in MLS, suggesting the link to round cell change. [6]. Guo et al. reported that PI-103, a dual PI3K/mTOR inhibitor, effectively inhibited the activation of the PI3K/Akt in liposarcoma cell lines and induced apoptosis. In addition, the combination of PI-103 with doxorubicin and cisplatin demonstrated strong synergized the growth-inhibitory effect [35]. These findings also suggest that the PI3K/Akt pathway could play a role in malignant phenotype formation of MLS.

This study has some limitations. First, this is a retrospective study with only a small number of patients enrolled and only one tumor having a >5 % round cell component. Second, there was heterogeneity in the treatment, including different modalities such as surgery, chemotherapy, radiotherapy, and their combinations. The treatment strategy varied depending on the presence of a round cell component, tumor size and location, and surgical margin.

## Conclusions

Our findings showed that high PPARγ expression was significantly associated with the presence of distant metastasis in patients with MLS. PPARγ expression may be a putative novel prognostic marker of MLS. Further investigations are necessary to confirm our findings and reveal the underlying mechanistic correlation between PPARγ and MLS malignancy.

## Abbreviations

CHOP, C/EBP homologous protein; DDIT3, DNA damage-inducible transcript 3; FUS, fused in sarcoma; GADD153, DNA damage-inducible gene 153; IGF1R, insulin like growth factor 1 receptor; IHC, immunohistochemistry; MLS, myxoid liposarcoma; mTOR, mechanistic target of rapamycin; PI3K, phosphoinositide 3-kinase; PI3KCA, phosphatidylinositol-4,5-bisphosphate 3-kinase catalytic subunit alpha; PPARγ, peroxisome proliferator-activated receptor gamma; PTEN, phosphatase and tensin homolog
